# Meta-analysis of risk factors associated with oxaliplatin hypersensitivity reactions in cancer patients

**DOI:** 10.1007/s10147-021-02034-3

**Published:** 2021-10-09

**Authors:** Linhui Zhu, Huan Li, Qiong Du, Xuan Ye, Sijia Yu, Xin Luo, Qing Zhai

**Affiliations:** 1 Department of Pharmacy, Fudan University Shanghai Cancer Center, Shangai, 200032 China; 2 Department of Oncology, Shanghai Medical College, Fudan University, Shanghai, 200032 China

**Keywords:** Oxaliplatin, Hypersensitivity reactions, Risk factors, Meta-analysis

## Abstract

This study aimed to investigate risk factors associated with oxaliplatin hypersensitivity reactions in cancer patients through a meta-analysis. A comprehensive retrieve of Chinese databases China National Knowledge Infrastructure, Wanfang Data, VIP Database and English databases PubMed, ScienceDirect, Embase and Cochrane library was conducted. The studies that meet the requirements for meta-analysis according to inclusion and exclusion criteria were screened and assessed for eligibility. Odds ratio (OR) / Weighted mean difference (WMD) and 95% confidence intervals (95% CIs) or calculable dichotomous and continuous raw data were extracted to perform meta-analysis using random effect model or fixed effect model on the basis of heterogeneity between studies through Review Manager 5.4 software. A total of 14 cross-sectional studies and 3367 cancer patients were included. Meta-analysis results showed that platinum exposure history (OR value 3.13, 95% CI 2.19–4.48, heterogeneity *P* = 0.26), allergy history (OR value 1.76, 95% CI 1.09–2.85, heterogeneity *P* = 0.61), platinum free interval (OR value 3.75, 95% CI 2.00–7.06, heterogeneity *P* = 0.83), dexamethasone premedication dose (OR value 0.28, 95% CI 0.13–0.58, heterogeneity *P* = 0.21) were significantly correlated to oxaliplatin hypersensitivity reactions. Gender, age, metastasis, combination with bevacizumab, XELOX regimen and cancer types were detected to have no statistically significant effect on oxaliplatin hypersensitivity reactions. Platinum exposure history, allergy history and long platinum-free interval are risk factors of oxaliplatin hypersensitivity reactions. High dexamethasone premedication dose is a protective factor of oxaliplatin hypersensitivity reactions.

## Introduction

Cancer has been a public health problem and the main cause of death in patients worldwide. In 2018 there were about 18 million new cancer cases worldwide and 9.6 million patients died [1], which had seriously affected human life quality. Platinum drugs, as the basic drugs in chemotherapy for cancer patients, are widely used in the treatment of a variety of malignant tumors. Oxaliplatin is the third-generation platinum cytotoxic compound, which contains a 1,2 diaminocyclohexane carrier and an oxalate-based ligand. As a broad-spectrum anti-tumor agent, oxaliplatin has been clinically used in the treatment of multiple cancers [2]. Oxaliplatin adverse reactions are mainly manifested in neurotoxicity, hematological toxicity and gastrointestinal toxicity, and apart from these reactions oxaliplatin may also cause hypersensitivity reactions in cancer patients [3]. Hypersensitivity reaction is defined as an unexpected reaction with signs and symptoms not consistent with known toxicity of the drug [4]. Common clinical manifestations of oxaliplatin hypersensitivity reactions are pruritus, skin rash, dizziness, diarrhea, bronchospasm, itching, blood pressure changes, etc. and moreover, severe reactions may even lead to death events [3]. In recent years as oxaliplatin application in adjuvant and palliative chemotherapy has increased, the incidence of hypersensitivity reactions has further risen up between 8.9 and 24.0% [5–9]. For some cancer patients, oxaliplatin has become a vital part of the chemotherapy regimen. If a severe hypersensitivity reaction occurs which interrupts chemotherapy process and oxaliplatin reintroduction, disease control may be delayed and overall survival may also be shortened. Therefore, as an important and non-negligible adverse reaction in clinical practice, investigating risk factors of oxaliplatin hypersensitivity reactions can help clinicians be aware of patients monitoring and clinical management. As current researches on risk factors of oxaliplatin hypersensitivity reactions have still not drawn unified conclusion, this study aims to systematically explore the association between possible risk factors and oxaliplatin hypersensitivity reactions by means of a meta-analysis.

## Materials and methods

### Search strategy

This meta-analysis was conducted on the basis of the meta-analysis of Observational Studies in Epidemiology (MOOSE) guidelines [10]. The literature search was conducted in Chinese databases China National Knowledge Infrastructure, Wanfang Data, VIP Database and foreign databases PubMed, ScienceDirect, Embase and Cochrane library, using combinations of the following key terms: (oxaliplatin) AND (hypersensitivity OR hypersensitivity reaction OR allergy OR allergic reaction) AND (risk factor). The full time period available for each database was searched up to February, 2021. Besides, references in searched relevant reviews were chosen for additional studies.

### Study selection

Eligible studies needed to meet the following inclusion criteria: (1) the study population was cancer patients; (2) the study contained risk factors of oxaliplatin hypersensitivity reactions; (3) the study design was observational study; (4) the study reported odds ratio (OR) / weighted mean difference (WMD) with 95% confidence interval (CI) or raw data which can calculate these values; (5) oxaliplatin hypersensitivity reactions needed to be graded according to severity; (6) the study published language was Chinese or English. Exclusion criteria were the following: (1) review or comment; (2) case report; (3) abstract without full text; (4) the study with incomplete or incorrect data; (5) repetitive study or same population; (6) the study did not contain outcome related with risk factors of oxaliplatin hypersensitivity.

### Data extraction and quality assessment

The following data were extracted from each study: (1) the first author’s name and year of publication; (2) country; (3) study design; (4) study population; (5) number of cancer patients; (5) mean age of population; (6) number of men; (7) chemotherapy drugs; (8) median cycle; (9) median oxaliplatin cumulative dose; (10) involved risk factors; (11) incidence of oxaliplatin hypersensitivity reactions; (12) study quality score. OR/WMD value and 95% confidence interval with multivariate analysis would be directly extracted from each study (if the study did not conduct multivariate analysis, the results of univariate analysis would be accepted) or calculated based on the incidence of oxaliplatin hypersensitivity reactions in risk factors group and control group.

The Newcastle–Ottawa Quality Assessment Scale (NOS) was used to assess case–control studies and cohort studies [11]. The risk of bias was low if the score was 6 or more and the risk of bias was high if the score was 5 or less [12]. Agency for Healthcare Research and Quality (AHRQ) Cross-Sectional/Prevalence Study Quality was used to assess cross-sectional studies [11], with a score of 0–3 for low-quality study, 4–7 for moderate-quality study and 8–11 for high-quality study, respectively [13].

### Statistical analysis

Review Manager 5.4 software was applied to conduct this meta-analysis of all included studies. The Q statistic test and *I*^2^ value were calculated to assess heterogeneity between studies [14, 15]. *P* < 0.1 and *I*^2^ > 50% was considered high heterogeneity among studies and random effect model would be used to perform the analysis. *P* ≥ 0.1 and *I*^2^ ≤ 50% was regarded low heterogeneity among studies and fixed effect model would be used to perform the analysis. Dichotomous outcomes were expressed as OR and 95% CI and continuous outcome were expressed as WMD and 95% CI with *P* < 0.05 considered to be statistically significant. Forest plots were applied to display outcomes graphically and the funnel plot was used to examine the potential publication bias [16]. Sensitivity analyses were performed by applying different models on each factor and omitting one study at a time among high-heterogeneity risk factor group to assess influence on pooled estimate results.

## Results

### Search results

**Fig. 1:**
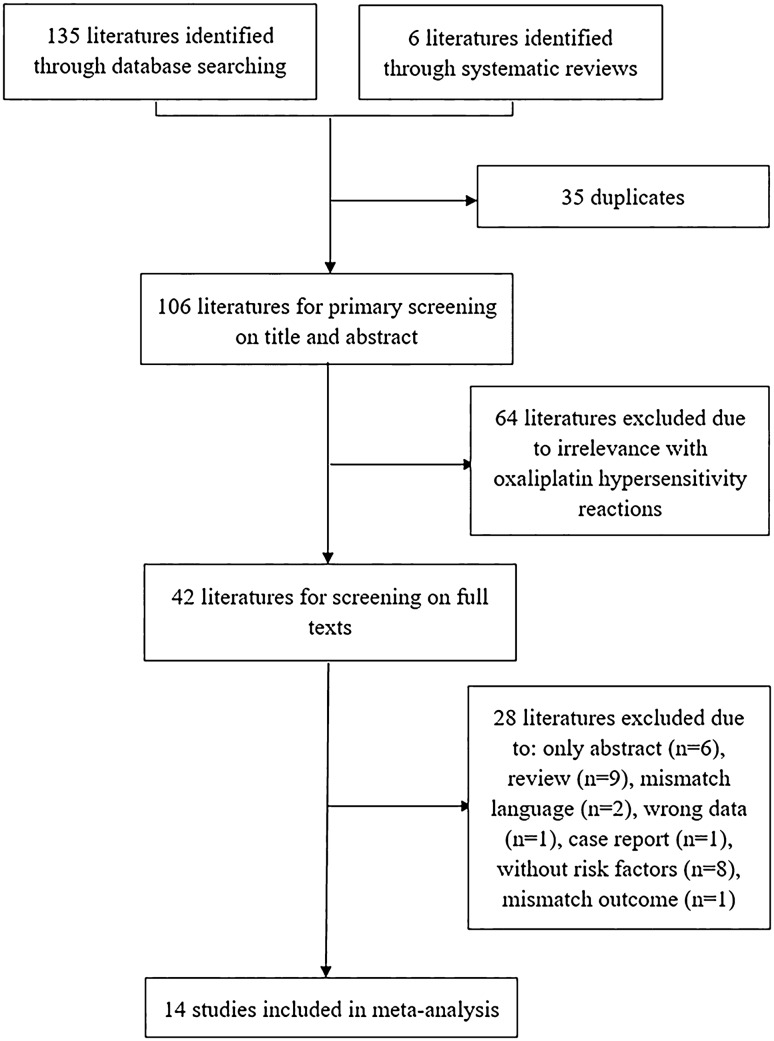
Flowchart of study search and selection

The literature search yielded a total of 141 potentially relevant articles, 35 of which were duplicate literatures. Sixty-four studies were excluded due to irrelevant research topics with oxaliplatin hypersensitivity reactions through primary screening on title and abstract of each literature. Of the remains, 28 literatures not meeting inclusion criteria were removed out and 14 studies which contained investigation of oxaliplatin hypersensitivity reactions were included, 4 of which were published in Chinese from China National Knowledge Infrastructure, Wanfang Data and VIP Database. The flowchart of the process on literature search and study selection was shown in Fig. 1.

### Study characteristics

Table 1 reported basic characteristics of 14 studies [17–30] in this meta-analysis. All studies included for analysis were cross-sectional studies, which were published after 2009. The number of cancer patients ranged from 62 to 793 (total *N* = 3367), with number of men patients from 35 to 481 and mean age from 51 to 66. Eight of the included studies were conducted in colorectal cancer patients, one was in gastrointestinal and urinary cancer patients and the other four were in all cancer patients with no restriction on cancer types. All studies were assessed for methodological quality with AHRQ Cross-Sectional/Prevalence Study Quality, 12 of which were moderate quality study and 2 of which were high-quality study. There were no studies with low-quality rating. Table 2 displays the outcome of oxaliplatin hypersensitivity reactions in each study. Cancer patients in studies received chemotherapy regimen containing oxaliplatin in combination with some of the following drugs: leucovorin, 5-*FU*, capecitabine, gemcitabine, *S*-1, docetaxel, etoposide, epirubicin, vindesine, raltitrexed, irinotecan, endostatin, bevacizumab, cetuximab, sunitinib and panitumumab. Oxaliplatin hypersensitivity reactions median chemotherapy cycle ranged from 5 to 9 with four studies no mention, while median cumulative oxaliplatin dose varied from 775.0 to 895.0 mg and from 400.9 to 625.7 mg*/*m^2^ with seven studies no mention. Risk factors associated with oxaliplatin hypersensitivity reactions in each study included gender, age, platinum exposure history, allergy history, metastasis, platinum-free interval, combination with bevacizumab, XELOX regimen, dexamethasone premedication dose and cancer types. The incidence of oxaliplatin hypersensitivity reactions remained between 4.9 and 22.2%, among which two Chinese studies reported lower hypersensitivity reactions incidence (4.9 and 6.6%) than current reviews [17, 20].

**Table 1:** Basic characteristics of included studies

Author, year	Country	Study design	Study population	Number of patients	Mean age	Number of men	AHRQ score
Li et al*.* 2018 [17]	China	Cross-sectional study	Colorectal cancer	242	60	156	7
Du et al*.* 2014 [18]	China	Cross-sectional study	Gastrointestinal and urinary cancer	194	55	144	5
Shen et al*. * 2013[19]	China	Cross-sectional study	Colorectal cancer	139	63	78	6
Zhu et al*.* 2017 [20]	China	Cross-sectional study	Colorectal cancer	320	51	177	6
Ohta et al*.* 2017 [21]	Japan	Cross-sectional study	Colorectal cancer	240	66	107	9
Mori et al*.* 2010 [22]	Japan	Cross-sectional study	Colorectal cancer	223	–	148	5
Kim et al*.* 2009 [23]	America	Cross-sectional study	All cancer	247	60	125	6
Okayama et al*.* 2015 [24]	Japan	Cross-sectional study	Colorectal cancer	162	64	88	7
Parel et al*.* 2014 [25]	France	Cross-sectional study	All cancer	119	62	78	7
Kim et al*. *2012 [26]	Korea	Cross-sectional study	All cancer	393	59	213	7
Sohn et al*.* 2018 [27]	Korea	Cross-sectional study	All cancer	793	59	481	7
Shibata et al. 2009 [28]	Japan	Cross-sectional study	Colorectal cancer	125	60	73	4
Yamauchi et al*.* 2015 [29]	Japan	Cross-sectional study	Colorectal cancer	62	63	35	4
Seki et al*.* 2011 [30]	Japan	Cross-sectional study	Colorectal cancer	108	64	67	8

**Table 2:** Oxaliplatin hypersensitivity outcomes of each study

Author, year	Chemotherapy drugs	Median chemotherapy cycle	Median cumulative oxaliplatin dose	Risk factors	Hypersensitivity reactions incidence/%
Li et al*.* 2018 [17]	abcdk	6	895.0 mg	④⑥⑧	4.9
Du et al*.* 2014 [18]	abcdefghjlm	7	478.7 mg /m^2^	①②④	10.8
Shen et al*.* 2013 [19]	abcdfl	7	775.0 mg	①⑨	10.1
Zhu et al*. * 2017 [20]	abcdk	8	625.7 mg /m^2^	①⑩	6.6
Ohta et al*. * 2017 [21]	abc	–	–	③⑤⑦	16.3
Mori et al*.* 2010 [22]	abc	–	–	①④⑥⑩	20.2
Kim et al*.* 2009 [23]	abcdek	7	–	①②⑦⑧⑩	11.7
Okayama et al*.* 2015 [24]	abcdkl	8	582.0 mg/m^2^	①②⑤⑧	17.2
Parel et al*.* 2014 [25]	abcdeiklno	5	400.9 mg/m^2^	①②③④	8.9
Kim MY et al*.* 2012 [26]	abcdkp	8	–	①⑨	10.7
Sohn et al*.* 2018 [27]	abcdfo	–	–	①③⑥	18.7
Shibata et al*.* 2009[28]	abc	9	–	①④	17.0
Yamauchi et al*.* 2015 [29]	abcdfkq	8	544.5 mg/m^2^	①②③⑤⑦⑨	11.3
Seki et al*.* 2011[30]	abc	–	–	①④	22.2

Chemotherapy drugs: a = oxaliplatin; b = leucovorin, c = 5-FU, d = capecitabine, e = gemcitabine, f = S-1, g = docetaxel, h = etoposide, i = epirubicin, j = vindesine, k = bevacizumab, l = cetuximab, m = endostatin, n = raltitrexed, o = irinotecan, p = sunitinib q = panitumumab

Risk factors: ① gender, ② age, ③ platinum exposure history, ④ allergy history, ⑤metastasis, ⑥ platinum free interval, ⑦ combination with bevacizumab, ⑧ XELOX regimen, ⑨ dexamethasone premedication dose, ⑩ cancer types

### Heterogeneity

This meta-analysis investigated ten risk factors related to oxaliplatin hypersensitivity reactions. Among them, gender (*I*^2^ = 52%, *P* = 0.02) and age (*I*^2^ = 82%, *P* = 0.0002) were analyzed through random effect model. Other risk factors including platinum exposure history (*I*^2^ = 25%, *P* = 0.26), allergy history (*I*^2^ = 0%, *P* = 0.61), metastasis (*I*^2^ = 0%, *P* = 0.47), platinum-free interval (*I*^2^ = 0%, *P* = 0.83), combination with bevacizumab (*I*^2^ = 0%, *P* = 0.86), XELOX regimen (*I*^2^ = 7%, *P* = 0.34), dexamethasone premedication dose (*I*^2^ = 35%, *P* = 0.21) and cancer type (colon cancer) (*I*^2^ = 0%, *P* = 0.46) were analyzed through fixed effect model.

## Risk factors associated with oxaliplatin hypersensitivity reactions

### Gender

**Fig. 2:**
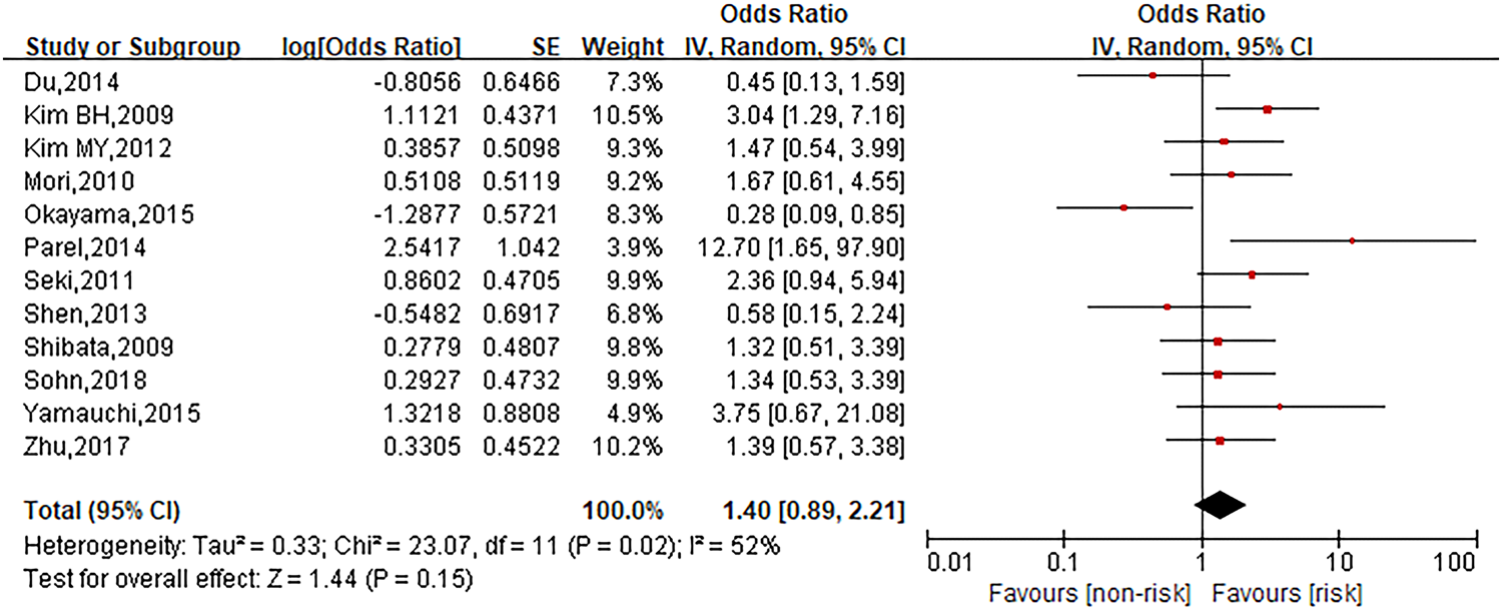
Forest plot of the association with gender and oxaliplatin hypersensitivity reactions

Twelve studies were involved into analysis to compare patients in female group with those in male group and the pooled OR for oxaliplatin hypersensitivity reactions was 1.40 (95% CI 0.89 ~ 2.21, *Z* = 1.44, *P* = 0.15) to show no statistically significant differences between both groups. The forest plot was displayed in Fig. 2.

### Age

**Fig. 3:**
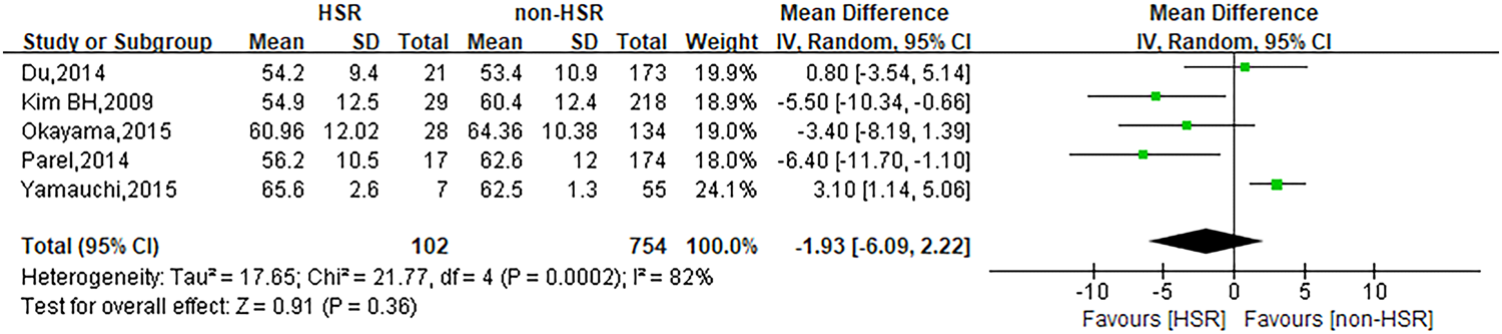
Forest plot of the association with age and oxaliplatin hypersensitivity reactions

Five studies were involved in the analysis to compare patients in hypersensitivity reaction group with those in non-hypersensitivity reaction group and the pooled WMD for age was −1.93 (95% CI −6.09 ~ 2.22, *Z* = 0.91, *P* = 0.36) to show no statistically significant differences between both groups. The forest plot is displayed in Fig. 3.

### Platinum exposure history

**Fig. 4:**
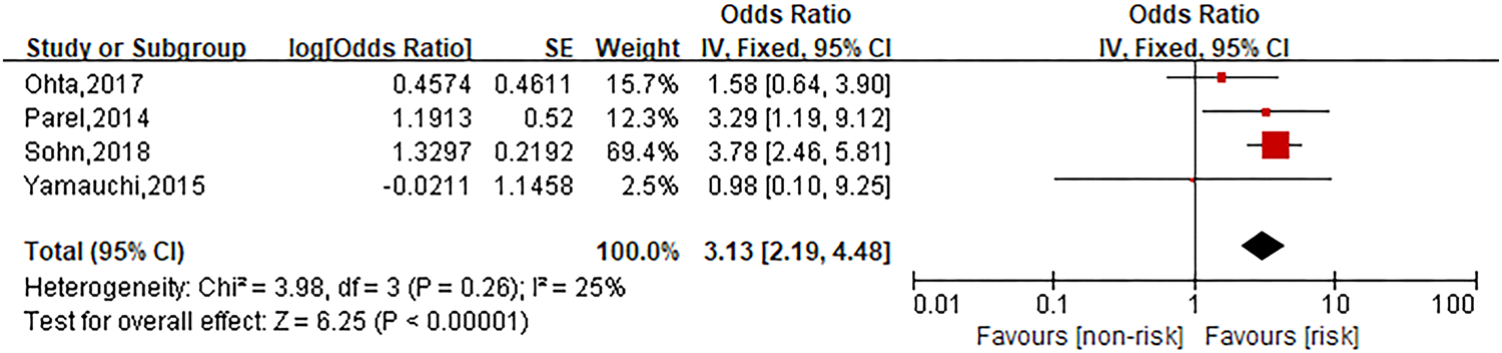
Forest plot of the association with platinum exposure history and oxaliplatin hypersensitivity reactions

Four studies were involved in the analysis to compare patients in platinum exposure history group with those in no platinum exposure history group and the pooled OR for oxaliplatin hypersensitivity reactions was 3.13 (95% CI 2.19–4.48, *Z* = 6.25, *P* < 0.00001) to show platinum exposure history considerably increased the risk of oxaliplatin hypersensitivity reactions. The forest plot is displayed in Fig. 4.

### Allergy history

**Fig. 5:**
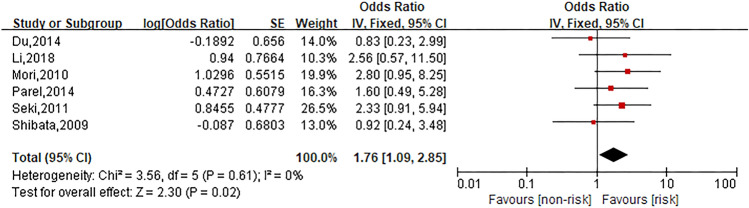
Forest plot of the association with allergy history and oxaliplatin hypersensitivity reactions

Six studies were involved in the analysis to compare patients in allergy history group with those in no allergy history group and the pooled OR for oxaliplatin hypersensitivity reactions was 1.76 (95% CI 1.09–2.85, *Z* = 2.30, *P* = 0.02) to show allergy history considerably increased the risk of oxaliplatin hypersensitivity reactions. The forest plot is displayed in Fig. 5.

### Metastasis

**Fig. 6:**
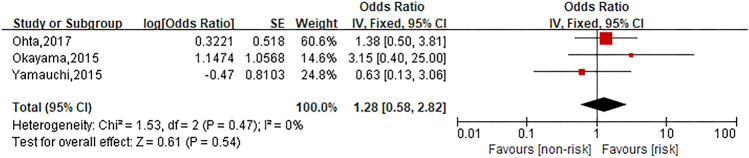
Forest plot of the association with metastasis and oxaliplatin hypersensitivity reactions

Three studies were involved in the analysis to compare patients in metastasis group with those in no metastasis group, and the pooled OR for oxaliplatin hypersensitivity reactions was 1.28 (95% CI 0.58–2.82, *Z* = 0.61, *P* = 0.54) that showed no statistically significant differences between both groups. The forest plot is displayed in Fig. 6.

### Platinum-free interval

**Fig. 7:**
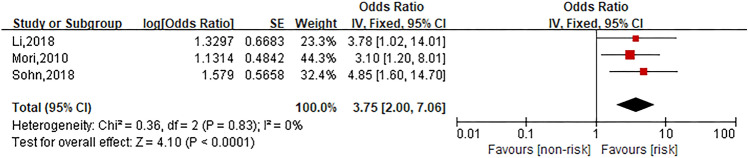
Forest plot of the association with long platinum free interval and oxaliplatin hypersensitivity reactions

Three studies were involved in the analysis to compare patients in long platinum free interval group with those in short platinum free interval group and the pooled OR for oxaliplatin hypersensitivity reactions was 3.75 (95% CI 2.00–7.06, *Z* = 4.10, *P* < 0.0001) that showed long platinum-free interval considerably increased the risk of oxaliplatin hypersensitivity reactions. The forest plot is displayed in Fig. 7.

### Combination with bevacizumab

**Fig. 8:**
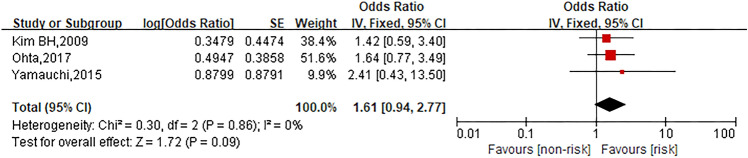
Forest plot of the association with combination with bevacizumab and oxaliplatin hypersensitivity reactions

Three studies were involved in the analysis to compare patients in bevacizumab group with those in no combination with bevacizumab group and the pooled OR for oxaliplatin hypersensitivity reactions was 1.61 (95% CI 0.94–2.77, *Z* = 1.72, *P* = 0.09) that showed no statistically significant differences between both groups. The forest plot is displayed in Fig. 8.

### XELOX regimen

**Fig. 9:**
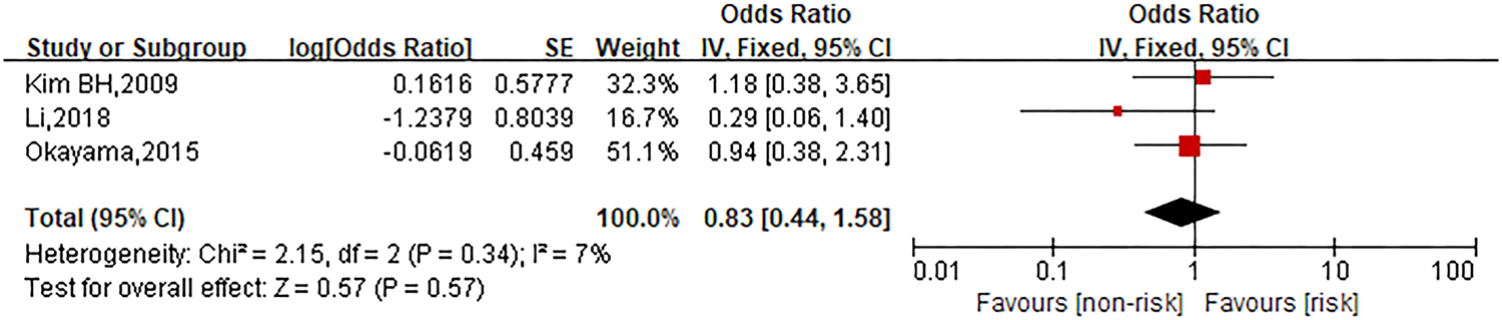
Forest plot of the association with XELOX regimen and oxaliplatin hypersensitivity reactions

Three studies were involved in the analysis to compare patients in XELOX regimen group with those in other regimen group and the pooled OR for oxaliplatin hypersensitivity reactions was 0.83 (95% CI 0.44–1.58, *Z* = 0.57, *P* = 0.57) that showed no statistically significant differences between both groups. The forest plot is displayed in Fig. 9.

### Dexamethasone premedication dose

**Fig. 10:**
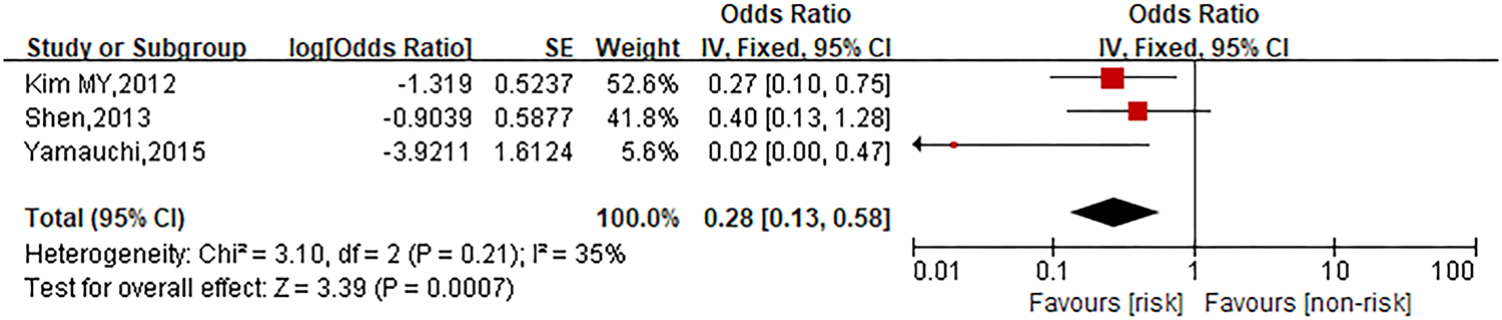
Forest plot of the association with high dexamethasone premedication dose and oxaliplatin hypersensitivity reactions

Three studies were involved in the analysis to compare patients in high dose group with those in low dose group and the pooled OR for oxaliplatin hypersensitivity reactions was 0.28 (95% CI 0.13–0.58, *Z* = 3.39, *P* = 0.0007) to show high dexamethasone premedication dose considerably decreased the risk of oxaliplatin hypersensitivity reactions. The forest plot is displayed in Fig. 10.

### Cancer type (colon cancer)

**Fig. 11:**
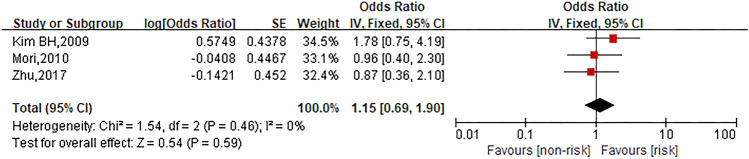
Forest plot of the association with colon cancer and oxaliplatin hypersensitivity reactions

Three studies were involved in the analysis to compare patients in colon cancer group with those in other cancer types group and the pooled OR for oxaliplatin hypersensitivity reactions was 1.15 (95% CI 0.69–1.90, *Z* = 0.54, *P* = 0.59) to show no statistically significant differences between both groups. The forest plot is displayed in Fig. 11.

## Publication bias

**Fig. 12:**
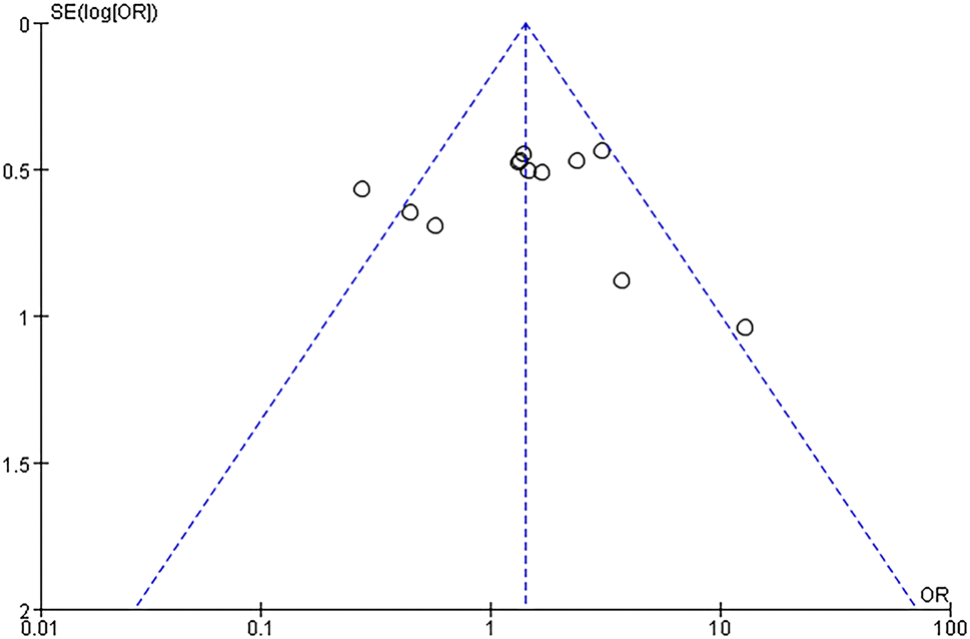
Funnel plot of gender factor

The funnel plot for gender risk factor with more than 10 studies was shown slight asymmetry that through visual inspection two studies deviated from the center of the funnel plot in Fig. 12.

## Sensitivity analysis

Another analysis model of each risk factor was used to perform analysis again and both results obtained from random effect model and fixed effect model were compared and exhibited in Table 3. Only conclusions drawn from two models of gender risk factor were different indicating obvious heterogeneity among studies, while other risk factors’ results from two models were similar.

**Table 3:** Pooled results of each risk factor with random effect model and fixed effect model

Risk factors	Random effect model	Fixed effect model
	OR/WMD [95% CI]	OR/WMD [95% CI]
Gender	1.40 [0.89, 2.21]	1.42 [1.04, 1.93]
Age	− 1.93 [− 6.09, 2.22]	0.55 [− 0.96, 2.07]
Platinum exposure history	2.85 [1.74, 4.66]	3.13 [2.19, 4.48]
Allergy history	1.76 [1.09, 2.85]	1.76 [1.09, 2.85]
Metastasis	1.28 [0.58, 2.82]	1.28 [0.58, 2.82]
Platinum free interval	3.75 [2.00, 7.06]	3.75 [2.00, 7.06]
Combination with bevacizumab	1.61 [0.94, 2.77]	1.61 [0.94, 2.77]
XELOX regimen	0.82 [0.42, 1.61]	0.83 [0.44, 1.58]
Dexamethasone premedication dose	0.25 [0.09, 0.69]	0.28 [0.13, 0.58]
Cancer type (colon cancer)	1.15 [0.69, 1.90]	1.15 [0.69, 1.90]

Risk factors with high heterogeneity of more than 50% were eliminated studies one by one and pooled analysis again. (1) Gender: After omitting the study by Okayama et al*.*, heterogeneity *I*^2^ decreased from 52 to 30% and the result changed. The pooled OR value was 1.61 (95% CI 1.09–2.38), suggesting that females may be more likely to develop oxaliplatin hypersensitivity reactions than males. (2) Age: After omitting the study by Yamauchi et al*.*, heterogeneity *I*^2^ decreased from 82 to 46% and the result changed. The pooled WMD value was −3.42 (95% CI −6.69 to −0.16), suggesting that younger patients may be more likely to occur oxaliplatin hypersensitivity reactions than elder patients.

## Discussion

Since oxaliplatin hypersensitivity reactions may interrupt basic chemotherapy of cancer patients, which puts adverse effects on disease control, it is crucial to identify risk factors of oxaliplatin hypersensitivity reactions early in clinical treatment to enhance patients’ management. Although current studies have analyzed risk factors of oxaliplatin hypersensitivity reactions, the opinions are still not unified and no unanimous conclusion can be reached. On the basis of previous studies, this meta-analysis assessed the affection of gender, age, platinum exposure history, allergy history, metastasis, platinum-free interval, combination with bevacizumab, XELOX regimen, dexamethasone premedication dose and cancer types (colon cancer) on oxaliplatin hypersensitivity reactions, among which platinum exposure history, allergy history, platinum-free interval and dexamethasone premedication dose are typical features that may have representative significance.

The pathophysiology of oxaliplatin hypersensitivity reactions remains not clarified yet, but most widely accepted mechanism is generally explained with IgE-mediated type I hypersensitivity. Degranulation of mast cells and basophils leading to nonimmune-mediated histamine and cytokines released arouses capillary expansion and smooth muscle contraction [5–7]. Clinical manifestations (rash, bronchospasm, hypotension, etc. during or immediately after oxaliplatin infusion), occurring time (after multiple oxaliplatin chemotherapy cycles), skin test positive and platinum-specific IgE test positive all support such pathophysiology [31]. Some rare symptoms such as hemolysis, arthralgia, proteinuria, etc. may be related to type II and III hypersensitivity reactions, involving tissue deposition of drug–antibody complexes and activation of the complement pathway [5, 9]. IgE-mediated hypersensitivity reactions usually occur after exposure to allergen once [32], so patients with allergy history may have produced specific IgE in vivo. Although studies do not elucidate previous allergens of patients with oxaliplatin hypersensitivity reactions, according to a research reported, exposure to antigens other than related drugs may lead to specific IgE to drugs, so that hypersensitivity reactions will occur with the first exposure to drugs [33]. At present this mechanism has not been verified in oxaliplatin, but it does provide possible theoretical support for oxaliplatin in patients with allergy history so that such patients need to be highly vigilant against hypersensitivity reactions’ occurrence if they infused oxaliplatin. Moreover, platinum exposure history differs from allergy history, patients with which experienced at least one exposure to platinum drugs, who may be stimulated with specific platinum IgE. Therefore, when they are exposed to oxaliplatin again, specific IgE activation leads to local or systemic hypersensitivity reactions. Sohn et al*.* [27] consider that in such patients hypersensitivity reactions are more likely to happen in an earlier chemotherapy cycle and show more severe symptoms, who require more cautious clinical management. In addition, some patients with exposure to oxaliplatin were medicated with oxaliplatin again after platinum free interval have increasing cumulative dose of oxaliplatin. Although patient's immune response weakened during platinum free interval, IgE will still be activated rapidly [17, 22]. Sohn et al*.* found that platinum-free interval of patients in oxaliplatin hypersensitivity group was significantly longer than that of patients in non-hypersensitivity group (21.6 vs. 16.8 months, *P* = 0.007), but the mechanism underlying longer platinum-free interval promoting hypersensitivity reactions is still unclear [27]. Therefore, for such patients with platinum-free interval, clinicians should evaluate risks of reintroduction and adopt premedication or desensitization to reduce the possibility of hypersensitivity reactions in patients.

Several studies report that premedication with steroids (e.g., dexamethasone, prednisone, methylprednisone) and antihistamines (e.g., ranitidine, diphenhydramine, promethazine) can reduce the occurrence of oxaliplatin hypersensitivity reactions to some extent [19, 34–36]. Shen et al*.* enrolled 139 colorectal cancer patients receiving oxaliplatin treatment and investigated that among 14 patients occurring hypersensitivity reactions, 3 of 5 patients receiving premedication with steroids and antihistamines successfully rechallenged oxaliplatin regimen, and premedication with dexamethasone was a potential risk factor of oxaliplatin hypersensitivity reactions [19]. Analogously, in order to assess certain rechallenge protocol targeting at patients with previous hypersensitivity reactions, Wu found 15 patients who experienced hypersensitivity reactions to oxaliplatin and underwent a rechallenge protocol containing premedication with dexamethasone, chlorpheniramine, ranitidine and all of them did not encounter another hypersensitivity reaction throughout the course of oxaliplatin treatment cycle [34]. A retrospective study by Lee et al*.* evaluated the efficacy of premedication for controlling oxaliplatin-related hypersensitivity reactions in patients with gastrointestinal malignancy, and of the 134 patients who were administered premedication with chlorpheniramine and hydrocortisone (totally 175 patients exhibiting hypersensitivity reactions to oxaliplatin), 71.6% had complete or partial prevention of hypersensitivity reactions after premedication [35]. Moreover, Lee et al*.* found that the success rate of completing oxaliplatin administration by premedication decreased as the severity of hypersensitivity reactions increased [35]. Sakaeda et al*.* reached a similar conclusion through data mining of the FDA Adverse Event Reporting System that dexamethasone administration effectively reduced oxaliplatin-induced mild hypersensitivity reactions, but had less impact on severe and lethal hypersensitivity reactions, while the effects of diphenhydramine to hypersensitivity reactions were examined no signals [36]. A prospective study by Yoshita et al*.* concluded that simultaneous infusion of oxaliplatin and dexamethasone increased pH of the infusion solution, thereby inhibiting the release of histamine and reducing the occurrence of hypersensitivity reactions [37]. Some studies have further concluded that higher pretreatment dose of steroids and antihistamines can lead to lower incidence of oxaliplatin hypersensitivity reactions. A retrospective cohort study by Kidera et al*.* reported that increased doses of dexamethasone and antihistamines significantly reduced incidence of oxaliplatin hypersensitivity reactions [38]. In our meta-analysis, Yamauchi et al*.* and Kim et al*.* believed that dexamethasone doses less than 12 and 20 mg, respectively, were risk factors for oxaliplatin hypersensitivity reactions [26, 29]. However, this premedication method does not work for all patients. Ohta et al*.* thought that dexamethasone premedication had nothing to do with oxaliplatin hypersensitivity [21]. In the study by Brandi et al*.*, six patients were administrated with dexamethasone premedication to reintroduce oxaliplatin, five of which still went through the same hypersensitivity reaction [39]. Many researchers discovered that quite a number of patients with oxaliplatin hypersensitivity reactions failed to rechallenge oxaliplatin-based chemotherapy even though they received premedication with steroids or antihistamines [40–42]. Currently, the selection and dosage of premedication drugs for oxaliplatin still lacks guidelines or high-level evidences to give standardized recommendations, so considerable work needs to be invested in the future to explore the impact of different premedication methods on oxaliplatin hypersensitivity reactions. In cases where the instructions and guidelines have not clearly indicated, clinicians generally choose premedication proposals based on personal experiences so that more attention should be paid on whether patients have hypersensitivity reactions even though with premedication.

This meta-analysis finds that age has nothing to do with oxaliplatin hypersensitivity reactions, but after excluding one study a positive conclusion is reached that younger patients are more likely to develop oxaliplatin hypersensitivity reactions. Heterogeneity among these studies attributes to the study by Yamauchi et al*.*, which almost drew the opposite conclusion that patients with hypersensitivity reactions to oxaliplatin were 3.1 years elder than those without hypersensitivity [29]. However, this study contained only 62 patients, whose small sample size led to high specificity and affected the accuracy of overall analysis. Previous studies have reported that infants, teenagers and the elderly were risk factors of anaphylaxis [43], but the mechanism as to how age influences oxaliplatin hypersensitivity is not yet known and, therefore, further exploration is required to verify cause and effect. Another risk factor with high heterogeneity, gender, is also found to have no relation with oxaliplatin hypersensitivity reactions in our meta-analysis. Identically, with excluding one study, heterogeneity obviously reduced and women was concerned as a risk factor of oxaliplatin hypersensitivity. Some researchers believe that women are more likely to develop drug hypersensitivity reactions [32, 43], the mechanism underlying which may ascribe to hormones [23], while the study by Okayama et al*.* which produced heterogeneity of gender indicated that men were more likely to occur oxaliplatin hypersensitivity reaction [24]. Based on the findings above, whether gender has a significant effect on oxaliplatin hypersensitivity reactions still needs to be further explored.

The following are the potential limitations in this study: (1) all cross-sectional studies are included with lack of case–control studies and cohort studies. Evidence level of literature keeps not high enough that may cause certain potential bias and lack of persuasiveness with the conclusion to some extent; (2) some of the risk factors analyzed involve less researches and thereby the pooled results are not reliable enough that more data are required to increase the stability of the evidence; (3) this study has a wider geographical variation with most patients included belong to Asians, of which two studies’ populations are from the United States and France, so certain publication bias exists. The individual differences in oxaliplatin hypersensitivity reactions actually remain, so big gap among the ethnicity of study population may exacerbate the clinical heterogeneity; (4) in this study only a risk factor with not less than three studies were analyzed. The remaining factors such as palliative chemotherapy, cumulative dose, eosinophils, albumin, etc. cannot be counted due to lack of data, on which some researches hold affirmative thoughts indeed with no explanation that they are irrelevant with oxaliplatin hypersensitivity reactions; (5) this study is a review of the current evidence without analyzing individual data. Based on the results, we assume that premedication is a point that is worth further exploration, so as the next step we are going to study the effect of different premedication regimens on oxaliplatin hypersensitivity in real-world settings.

In summary, our meta-analysis shows that platinum exposure history, allergy history and long platinum-free interval are important risk factors of oxaliplatin hypersensitivity reactions and high dexamethasone premedication dose is a protective factor of oxaliplatin hypersensitivity. The danger of hypersensitivity to oxaliplatin should not be underestimated This study may help identify patients with risk factors and conduct clinical monitoring and management so as not to affect patients’ chemotherapy process and life quality. Additional prospective studies with larger sample size and high-level evidence are needed to further investigate risk factors associated with oxaliplatin hypersensitivity reactions.

## Data Availability

No additional data are available.
